# VEGF Is a Stronger Predictor of Depressive Symptoms than Other Inflammation Markers in People with HIV on Antiretroviral Therapy

**DOI:** 10.3390/v18060628

**Published:** 2026-05-30

**Authors:** Dana Gabuzda, Jun Yin, Hajime Uno, Susan Morgello

**Affiliations:** 1 Department of Cancer Immunology and Virology, Dana-Farber Cancer Institute, Harvard Medical School, Boston, MA 02215, USA; 2 Department of Neurology, Harvard Medical School, Boston, MA 02115, USA; 3 Department of Data Science, Dana-Farber Cancer Institute, Harvard Medical School, Boston, MA 02215, USA; 4 Department of Neurology, Icahn School of Medicine at Mount Sinai, New York, NY 10029, USA

**Keywords:** HIV, biomarkers, comorbidities, depression, inflammation, VEGF

## Abstract

People with HIV (PWH) experience higher rates of depression compared with the general population. Inflammation has been associated with depressive symptoms and may be associated with a subtype of depression, but the relationship between inflammation and depressive symptoms in PWH on antiretroviral therapy (ART) is unclear. In this study, inflammation biomarkers (IFN-γ, IL-1β, IL-6, IL-8, IL-12p70, IL-15, IP-10, MCP-1, VEGF, CRP) were measured in plasma from 195 PWH on ART and depressive symptoms were assessed using Beck Depression Inventory-II (BDI-II) scores. Logistic regression and mixed-effects models were used to examine the associations between inflammation biomarkers and depressive symptoms at baseline and over 18 months of follow-up. PWH had a median age of 52 years, with 95% being virally suppressed below 200 copies/mL, 33% having high depressive symptoms, and 62% having ≥1 medical comorbidity (HCV, cardiovascular disease, diabetes, chronic kidney disease, chronic lung disease). VEGF levels were increased in PWH with high vs. low depressive symptoms (*p* = 0.01). The association between VEGF and depressive symptoms remained significant in covariate-adjusted models (*p* = 0.005) and was augmented in PWH with medical comorbidities (*p* = 0.002). Other inflammation biomarkers were increased in PWH with medical comorbidities, but not significantly different between groups stratified by depressive symptoms. Among PWH with high depressive symptoms, biomarker clustering identified inflammatory and noninflammatory subgroups distinguished by levels of VEGF/MCP-1/IL-8 or IL-6/CRP and prevalence of HCV, cardiovascular disease, and diabetes. These findings suggest that VEGF is a biomarker associated with depressive symptoms in PWH on ART and may identify a subtype of depression for targeted interventions.

## 1. Introduction

People with HIV (PWH) have an increased prevalence of depression compared with the general population, with estimates ranging from 20% to 50% depending on the study population [1,2,3,4]. Several biological and psychosocial factors contribute to an increased risk of depression in PWH, including peripheral and central nervous system (CNS) effects of ongoing viral replication, chronic immune activation and inflammation, hypothalamic–pituitary–adrenal (HPA) axis dysregulation, some of the drugs used in antiretroviral therapy (ART), substance use, and chronic stress [5,6,7,8,9]. Depression in PWH has been associated with delayed ART initiation, reduced ART adherence, and increased morbidity and mortality [3,10,11,12]. Thus, understanding the etiologies and biological mechanisms of depression in PWH and delivering effective interventions is important for maintaining viral suppression and improving health outcomes.

Inflammation has been associated with depressive symptoms in the general population [13,14] and may be linked to an inflammatory subtype of depression in PWH [1,15,16,17]. Inflammation in PWH can alter the metabolism of tryptophan and neurotransmitters implicated in depression, particularly serotonin and dopamine [18,19,20,21,22]. Inflammation can also trigger dysregulation of the HPA axis in PWH, leading to depression through dysregulation of cortisol and neurosteroids [23,24]. While some studies detected positive associations between inflammation markers (e.g., IL-1β, IL-6, TNF-α, CRP) and depressive symptoms in PWH on ART [16,17,25], other studies detected an association between inflammation markers and depression only in men [26,27] or no significant association [28,29]. Explanations for the heterogeneous findings across studies on associations between inflammation biomarkers and depression in PWH remain unclear [7,9,10].

The identification of biomarkers that can distinguish between inflammatory and noninflammatory subtypes of depression and their underlying mechanisms in PWH is important for developing tailored interventions [1,13,14,30]. In this study, we examine the associations between inflammation biomarkers and depressive symptoms in cross-sectional and longitudinal analyses of PWH on ART with and without medical comorbidities.

## 2. Materials and Methods

### 2.1. Study Participants

Data and plasma samples from 260 eligible individuals (*n* = 195 HIV+ individuals on ART and *n* = 65 HIV-negative controls) were collected between 2006 and 2019. HIV+ individuals were enrolled at four sites in the National NeuroAIDS Tissue Consortium (NNTC) [31] and six sites in the CNS HIV Anti-Retroviral Therapy Effects Research (CHARTER) study [32]. Participants in these observational cohorts undergo standardized visits with formal assessments of HIV-related parameters, medical and neuropsychiatric conditions, medication use, and other clinical and lab data at 6- or 12-month intervals. All individuals were enrolled with written informed consent and institutional review board (IRB) approval at each study site. Eligible participants were adults between the ages of 30 and 75 years; PWH were taking 3 or more ART drugs for at least 1 year, with a formal assessment of depressive symptoms at the time of plasma sampling. A subset of PWH (*n* = 140) had 1 or more additional assessments of depressive symptoms within 18 months of follow-up. Plasma HIV RNA was measured at the time of plasma sampling. Undetectable plasma HIV RNA values were assigned a value of 40 copies/mL, reflecting the sensitivity cutoff of the assay most widely used during these assessments (50 copies/mL). Plasma samples from HIV− individuals without a diagnosed neurological disease or depressive disorder were from NNTC and Bioreclamation LLC (Westbury, NY, USA).

### 2.2. Outcome and Covariate Definitions

The primary outcome was depressive symptoms assessed using the Beck Depression Inventory-II (BDI-II, hereafter referred to as BDI score); high and low depressive symptoms were defined as a BDI score ≥ 14 and <14, respectively [33]. Cognitive function was assessed using a standardized neuropsychological test battery to assess seven cognitive domains; demographically corrected global neurocognitive T scores were derived by averaging seven cognitive domain T scores, as described [32,34]. HIV-associated neurocognitive disorder (HAND) diagnoses of asymptomatic neurocognitive impairment (ANI) or mild neurocognitive disorder (MND) (mild cognitive impairment with no interference or mild interference with everyday functioning, respectively) or HIV-associated dementia (HAD) (severe cognitive impairment) were determined using established criteria [35] based on neurocognitive testing and neurological evaluation. The following medical comorbidities were assessed based on self-report, medical records, and a review of medications and lab values, as described [36,37]: Hepatitis C virus (HCV) antibody positive, cardiovascular and cerebrovascular disease (CVD), diabetes, chronic kidney disease (CKD) stage 3–4, and chronic obstructive pulmonary disease (COPD).

### 2.3. Measurement of Inflammation Biomarkers

Ten inflammation biomarkers (IFN-γ, IL-1β, IL-6, IL-8, IL-12p70, IL-15, IP-10, MCP-1, VEGF-A [vascular endothelial growth factor, also known as VEGF], CRP) were measured in plasma samples using the Meso Scale Discovery (MSD) platform (Rockville, MD, USA) according to the manufacturer’s instructions, as described in previous studies [37,38]. These biomarkers were selected based on biological relevance to the immune pathophysiology of HIV and inflammation-associated comorbidities, including depressive disorders [16]. When biomarker levels were undetectable, samples were assigned the lowest detected value.

### 2.4. Statistical Analysis

All analyses were performed in R version 4.2.1. Cross-sectional analyses were conducted using the Mann–Whitney U test or Kruskal–Wallis test for continuous variables and a Chi-square test for categorical variables. Pearson’s correlation analyses were conducted to examine correlations between continuous variables. Of primary interest was to explore the possible associations between biomarkers and BDI scores and to examine whether plasma VEGF was more strongly associated with BDI scores than the other markers examined. Thus, *p*-values were not adjusted for multiple comparisons. Logistic regression models were used to assess the associations between biomarkers of interest and depressive symptoms among all PWH (*n* = 195) or subsets with or without medical comorbidities (*n* = 120 and *n* = 75, respectively) using the stats package. All multivariable models were adjusted for baseline age, sex, BMI, and antidepressant use. Longitudinal mixed-effects models, including random intercept and random slope, were fitted for VEGF as the independent variable and BDI scores as the dependent variable, with adjustment for covariates and time (months in study) using the lme4 package. Interaction terms for VEGF tertiles (high vs. middle/low tertile as a binary categorical variable) with time (months in study) were nonsignificant and, therefore, not used in the final models. K-means clustering was conducted for 10 inflammation biomarkers in PWH with depressive symptoms (*n* = 63) using the stats package. The optimal number of clusters was determined by the Silhouette method using the factoextra package.

## 3. Results

### 3.1. Characteristics of the Study Cohort

Included in the study were 195 HIV+ individuals on ART and 65 HIV− controls. HIV+ individuals were further categorized according to low or high depressive symptoms (*n* = 131 and *n* = 64, respectively) based on BDI score < 14 and ≥14, respectively, while HIV− individuals with depressive symptoms or depression diagnoses were excluded from the study. Characteristics of the study cohort by HIV status and depressive symptoms are shown in Table 1. HIV+ individuals had a median age of 52 years, 95% were virally suppressed below 200 HIV RNA copies/mL, 33% had high depressive symptoms, and 62% had ≥1 medical comorbidity (HCV coinfection, CVD, diabetes, chronic kidney disease, chronic lung disease). HIV+ vs. HIV− individuals were younger (median age 52 vs. 58 years for HIV+ and HIV−, respectively), with more males (85% vs. 60%) and lower BMI (median BMI 25 vs. 30), while proportions of current smokers (54% vs. 68%) and cocaine users (14% vs. 15%) were similar. Among 10 inflammation biomarkers measured in the study, IL-8, IL-15, IP-10, MCP-1, VEGF, and CRP levels were increased in HIV+ vs. HIV− individuals, while IL-1β, IFN-γ, IL-6, and IL-12p70 levels were similar.

HIV+ individuals with high vs. low depressive symptoms were similar with respect to age, race, sex, tobacco smoking, cocaine use, and HIV-related parameters, including duration of HIV infection, viral suppression below 200 copies/mL, and CD4+ T cell count (Table 1). However, there were differences in the prevalence of HAND diagnoses between HIV+ individuals with high vs. low depressive symptoms (*p* = 0.008), with a higher prevalence of HAD (8% vs. 0%, respectively) and MND (19% vs. 14%), and a lower prevalence of ANI (14% vs. 26%), but similar prevalence of normal cognitive function (48% vs. 48%). We observed non-significant trends for a higher prevalence of HCV seropositivity (36% vs. 26%), CVD (31% vs. 21%), CKD (13% vs. 8%), and COPD (27% vs. 17%) among HIV+ individuals with high vs. low depressive symptoms, while the prevalence of diabetes was similar (11% vs. 15%). When considering all five medical comorbidities, the prevalence of ≥1 medical comorbidity was slightly higher in HIV+ individuals with high vs. low depressive symptoms (72% vs. 57%, respectively; *p* = 0.055).

We compared inflammation biomarker levels between groups stratified by depressive symptoms (Table 1). Among inflammation biomarkers, only VEGF (median [IQR]: 64 [34–100] vs. 45 [31–66] pg/mL; *p* = 0.009) and MCP-1 (110 [86–128] vs. 97 [73–119] pg/mL; *p* = 0.041) levels were significantly increased in HIV+ individuals with high vs. low depressive symptoms, while other inflammation biomarker levels were similar (*p*-values > 0.10). When comparing inflammation biomarker levels between groups by medical comorbidity status, IL-6 (median [IQR]: 1.2 [0.8–2.3] vs. 1.0 [0.7–1.4] pg/mL; *p* = 0.031) and IL-15 (median [IQR]: 5.1 [3.8–7.5] vs. 4.6 [3.4–6.1] pg/mL; *p* = 0.0495) levels were increased in HIV+ individuals with vs. without medical comorbidities, respectively, while IP-10 exhibited an increasing trend (*p* = 0.098) and IL-1β, IFN-γ, IL-8, IL-12p70, MCP-1, VEGF, and CRP showed no significant difference (*p*-values > 0.15). Thus, the higher prevalence of medical comorbidities in HIV+ individuals with high vs. low depressive symptoms did not account for group differences in VEGF levels.

### 3.2. Association Between VEGF and Depressive Symptoms Is Augmented in HIV+ Individuals with Medical Comorbidities

Given the differences in VEGF and MCP-1 levels between groups stratified by depressive symptoms (Table 1), we examined these associations in logistic regression models fitted with log10 VEGF and MCP-1 as independent variables and depressive symptoms as the dependent variable. In univariate models, log10 VEGF (OR 4.02, 95% CI 1.47–11.6; *p* = 0.008), BMI (OR 0.88, 95% CI 0.81–0.95; *p* = 0.001) and antidepressant use (OR 2.79, 95% CI 1.52–5.21; *p* = 0.001) were associated with high depressive symptoms (Table 2). Log10 VEGF remained independently associated with high depressive symptoms after adjusting for age, sex, BMI, and antidepressant use (OR 4.97, 95% CI 1.65–15.83; *p* = 0.005) (Table 2). Interaction terms including log10 VEGF and sex or antidepressant use were not significant in these models.

Log10 MCP-1 was associated with depressive symptoms in univariate models and multivariable models adjusted for age, sex, BMI, and antidepressant use (Supplementary Table S1). However, when log10 VEGF was included as an additional term in these multivariable models, the association between MCP-1 and depressive symptoms was no longer significant. Thus, although log10 MCP-1 and log10 VEGF were positively correlated with each other among all 195 HIV+ individuals or a subset of 64 HIV+ individuals with high depressive symptoms (r = 0.26, *p* < 0.001; and r = 0.34, *p* = 0.006, respectively) (Supplementary Figure S1), VEGF, but not MCP-1, was an independent predictor of depressive symptoms.

A significant association between ≥1 medical comorbidity and high depressive symptoms was detected in the logistic regression models adjusted for age, sex, BMI, and antidepressant use (OR 2.0, 95% CI 1.02–4.00; *p* = 0.047) (Supplementary Table S2). When log10 VEGF was added to the model, both medical comorbidities and log10 VEGF remained independently associated with depressive symptoms (OR 2.1, 95% CI 1.08–4.41; *p* = 0.032 and OR 5.31, 95% CI 1.76–16.99; *p* = 0.004, respectively) (Supplementary Table S2). An interaction term between medical comorbidities and log10 VEGF was tested but was not significant. These findings suggest that medical comorbidities and VEGF had independent effects on depressive symptoms.

We stratified 195 HIV+ individuals into subgroups with or without ≥1 medical comorbidity and ran the same multivariable logistic regression model separately for each subgroup. The odds ratio of log10 VEGF as a predictor of high depressive symptoms in multivariable models including 120 HIV+ individuals with medical comorbidities (OR 7.63, 95% CI 2.16–30.24; *p* = 0.002) was higher than the same model including 75 HIV+ individuals without medical comorbidities (OR 2.19, 95% CI 0.19–27.07; *p* = 0.529) (Table 2). Thus, the association between VEGF and depressive symptoms was augmented in HIV+ individuals with medical comorbidities.

### 3.3. Longitudinal Association Between VEGF and BDI Score Is Augmented in HIV+ Individuals with Medical Comorbidities

**Figure 1:**
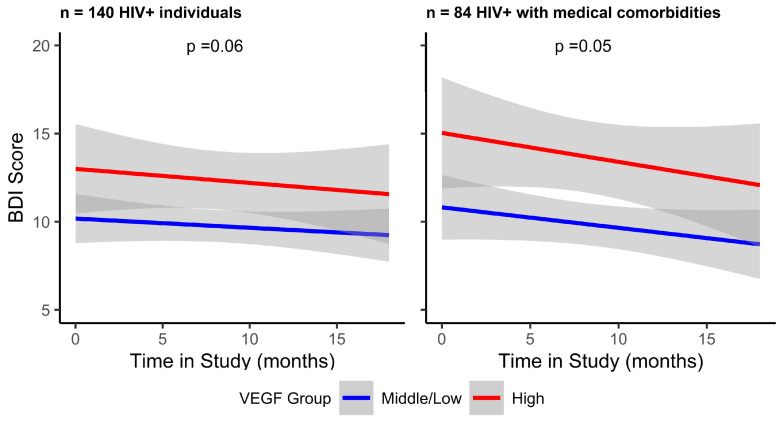
Longitudinal association between VEGF tertile and BDI scores is augmented in HIV+ individuals with medical comorbidities. Predicted values of BDI scores by VEGF tertile from longitudinal mixed-effects models adjusted for baseline age, sex, BMI, and antidepressant use among 140 HIV+ individuals (left panel) or 84 HIV+ individuals with 1 or more medical comorbidities (right panel) and 2 or more BDI scores within 18 months of follow-up. High VEGF tertile is associated with higher predicted mean BDI scores in both models. When individuals with high VEGF tertile had medical comorbidities, the effect of VEGF on BDI scores was augmented. Model statistics are shown in Table 3. *p*-values, fixed effects coefficient of VEGF tertile groups.

To further examine the association between VEGF levels and BDI scores, we built mixed-effects models to assess the longitudinal association between baseline VEGF tertile and estimated BDI score over 18 months of follow-up (*n* = 140 HIV+ individuals, *n* = 84 with and *n* = 56 without ≥1 medical comorbidity) (Table 3 and Figure 1, left panel). VEGF levels were modeled as a binary categorical variable based on VEGF tertiles (high vs. middle/low tertiles, corresponding to ≥65 and <65 pg/mL, respectively). In a univariate mixed-effects model including 140 HIV+ individuals and adjusting for time, the high VEGF tertile was associated with a higher BDI score (estimate = 3.47, *p* = 0.039). In a multivariable mixed-effects model adjusted for age, sex, BMI, antidepressant use, and time, the association between high VEGF tertile and BDI scores remained borderline significant (estimate = 3.19, *p* = 0.056). Interaction terms between VEGF high vs. middle/low tertile and time were nonsignificant when added to the model.

To further evaluate the effect of medical comorbidities on the longitudinal association between VEGF tertile and BDI scores, we tested separate mixed-effect models for HIV+ individuals with and without medical comorbidities (*n* = 84 and *n* = 56, respectively). In HIV+ individuals with medical comorbidities, the high vs. middle/low VEGF tertile was associated with a higher BDI score in the models adjusted for time (estimate = 4.08, *p* = 0.050) (Table 3 and Figure 1, right panel). In contrast, there was no significant association between VEGF tertile and BDI score in HIV+ individuals without medical comorbidities (Table 3). The difference of predicted mean BDI scores between the high vs. middle/low VEGF tertile was greater in longitudinal mixed-effects models including 84 HIV+ individuals with medical comorbidities compared with models including all 140 HIV+ individuals, providing further evidence that the effects of high VEGF tertile on BDI score were augmented in the setting of medical comorbidities.

### 3.4. Biomarker Clustering Separates HIV+ Individuals with High Depressive Symptoms into Clusters Distinguished by Inflammation Profiles, Comorbidities, and BDI Score Trajectories

**Figure 2:**
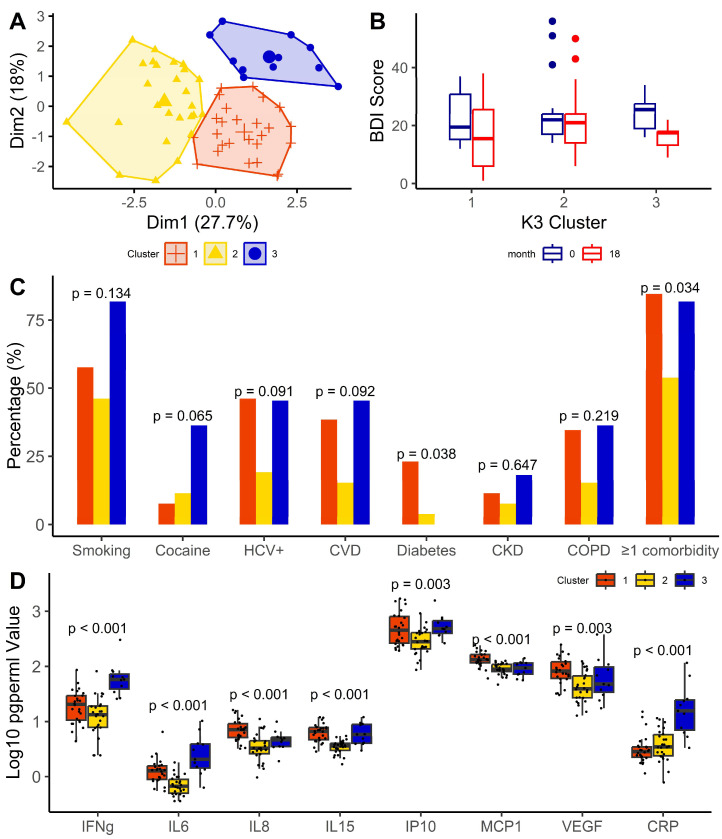
Biomarker clustering separates HIV+ individuals with high depressive symptoms into clusters distinguished by inflammation profiles, comorbidities, and change in BDI score over time. (**A**) Scatterplot shows dimensionality reduction of 10 inflammation biomarkers by principal component analysis (PCA) combined with K-means clustering, which separated 63 HIV+ individuals with high depressive symptoms into three distinct clusters. Each point represents an individual; clusters are color-coded red (*n* = 26), yellow (*n* = 26), and blue *n* = 11). The 1st and 2nd principal components (Dim1 and Dim2) explain 27.7% and 18.0% of the total variance, respectively. Contributions of each biomarker to Dim1 and Dim2 are in Supplementary Figure S2. (**B**) Paired plots of baseline vs. 18-month BDI scores for 33 HIV+ individuals with high depressive symptoms and paired values available at both time points according to biomarker-defined clusters (*n* = 10, *n* = 17, and *n* = 6 are represented in clusters 1, 2, and 3, respectively). Horizontal bars represent medians, boxes span the interquartile range (IQR), and whiskers extend to extreme data points. BDI, Beck Depression Inventory-II (BDI-II). (**C**) Bar plots show differences in frequency of smoking, cocaine use, or medical comorbidities among 63 HIV+ individuals with high depressive symptoms according to biomarker-defined clusters. Because the 10 biomarkers were used to define the clusters by K-means, the across-cluster differences in biomarker levels reflect the clustering input rather than independent statistical evidence; *p*-values are descriptive only. *p*-value, Chi-square test. HCV, hepatitis C virus; CVD, cardiovascular disease; CKD, chronic kidney disease; COPD, chronic obstructive pulmonary disease. (**D**) Levels of inflammation biomarkers significantly associated with biomarker-defined clusters for 63 HIV+ individuals with high depressive symptoms. See Supplementary Table S3 for details. Boxplots show log10-transformed biomarker levels according to three biomarker-defined clusters. Horizontal bars represent medians, boxes span the interquartile range (IQR), and whiskers extend to extreme data points. *p*-value, Kruskal–Wallis test. Units for CRP are µg/mL.

To explore the biomarker profiles that may distinguish between inflammatory and noninflammatory depressive subtypes described in the literature [13,14,30], we used an unsupervised clustering approach to group individuals with similar biomarker profiles and determine if these groups showed differences in clinical features. Biomarker clusters were derived by K-means clustering based on 10 inflammation biomarkers in 63 HIV+ individuals with high depressive symptoms after omitting one outlier (Figure 2). The optimal number of clusters was determined by the silhouette method (K = 3). Dimensionality reduction by principal component analysis (PCA) was used to transform and plot the biomarker data onto two dimensions (Dim1 and Dim2), which explained 28% and 18% of the total variance, respectively, mainly driven by IL-15, IL-6, IP-10, and IFN-γ (Dim1) and MCP-1, IFN-γ, VEGF, CRP, IL-8, and IL-12p70 (Dim2) (Supplementary Figure S2). A comparison of Dim1 and Dim2 showed good differentiation between the three clusters (Figure 2A).

The biomarker and clinical characteristics according to each cluster are shown in Figure 2 and Supplementary Table S3. Although all three clusters had high depressive symptoms (BDI score ≥ 14), clusters 1 and 3 tended to have higher BDI scores compared with cluster 2 (median BDI score 23 vs. 19 vs. 26 for clusters 1, 2, and 3, respectively). With regard to HAND diagnoses, cluster 1 had the highest proportion with MND (27% vs. 15% vs. 0%) and NPI-O (15% vs. 8% vs. 9%), while cluster 3 had the highest proportion with HAD (0% vs. 0% vs. 46%) and ANI (12% vs. 15% vs. 18%) and cluster 2 had the highest proportion with normal cognitive function.

To investigate if the trends of BDI scores over time were different between the biomarker-defined clusters, we evaluated paired BDI scores between baseline and 18 months among 33 individuals with high depressive symptoms at baseline and available BDI scores at both time points. The decline in BDI scores at 18-months compared to the baseline was more substantial in clusters 1 and 3 compared with cluster 2 (Figure 2B) (*p* = 0.028, *p* = 0.036, and *p* = 0.116, respectively, by paired Wilcoxon signed-rank test), suggesting differences in the longitudinal trajectories of depressive symptoms. In particular, the two clusters with higher VEGF levels showed more improvement in depressive symptoms compared to the cluster with lower VEGF levels over the 18-month observation period.

We examined the associations of biomarker-defined clusters with current smoking, cocaine use, and medical comorbidities (Figure 2C). Cluster 3 showed an increasing trend for more smokers (58% vs. 46% vs. 82% for clusters 1, 2, and 3, respectively) and cocaine users (8% vs. 12% vs. 36%) compared with the other clusters. Clusters 1 and 3 had more HCV (26% vs. 19% vs. 46% for clusters 1, 2, and 3, respectively; *p* = 0.09), CVD (39% vs. 15% vs. 46%; *p* = 0.09), CKD stage 3–4 (12% vs. 8% vs. 18%; *p* = 0.65), and COPD (35% vs. 15% vs. 36%; *p* = 0.22) compared with cluster 2, while the prevalence of diabetes was higher in cluster 1 vs. clusters 2 and 3 (23% vs. 4% vs. 0%; *p* = 0.04). Thus, the overall proportions with ≥1 medical comorbidity were higher in clusters 1 and 3 compared with cluster 2 (85% vs. 54% vs. 82%; *p* = 0.03), with the differences largely driven by more HCV, CVD, and diabetes in these clusters.

Within the scope of our investigation, 8 of 10 inflammation biomarkers showed significant differences when compared across biomarker-defined clusters (Figure 2D and Supplementary Table S3). Higher levels of inflammation biomarkers distinguished clusters 1 and 3 from cluster 2. Cluster 3 had the highest levels, followed by cluster 1, while cluster 2 had the lowest levels. Cluster 3 was distinguished by higher IL-6 and CRP levels compared with clusters 1 and 2, while cluster 1 was distinguished by higher VEGF, IL-8, and MCP-1 levels compared with clusters 2 and 3 (Supplementary Table S3). These results identify two patterns of inflammation biomarkers among HIV+ individuals with high depressive symptoms, distinguished by higher VEGF/MCP-1/IL-8 or IL-6/CRP levels and a higher prevalence of associated medical comorbidities, particularly HCV, CVD, and diabetes, and a third pattern characterized by no distinctive biomarker elevations and a lower prevalence of medical comorbidities.

## 4. Discussion

In this study, we explored the relationship between plasma inflammation markers and depressive symptoms in 195 virally suppressed PWH on ART and found that VEGF was a stronger predictor of depressive symptoms than the other inflammation markers examined. The association between VEGF and depressive symptoms was augmented in PWH with medical comorbidities and remained significant in cross-sectional and longitudinal models adjusted for age, sex, BMI, and antidepressant use. Other plasma inflammation markers were increased in PWH (e.g., IL-8, IL-15, IP-10, MCP-1, CRP) or the subset with medical comorbidities (e.g., IL-6, IL-15, IP-10) but had no significant association with depressive symptoms. Among PWH with high depressive symptoms, biomarker clustering identified two inflammatory subgroups distinguished by increased levels of VEGF/MCP-1/IL-8 or IL-6/CRP and prevalence of HCV, cardiovascular disease, and diabetes, and a third subgroup characterized by no distinctive biomarker elevations and lower prevalence of medical comorbidities. These findings suggest that VEGF is a biomarker associated with depressive symptoms in a subgroup of PWH on ART and may identify a biological subtype of depression.

While previous studies identified increased plasma or serum VEGF as a biomarker associated with major depressive disorder (MDD) in the general population [39,40,41,42,43,44], this study is the first to detect an association between VEGF and depressive symptoms in PWH. VEGF is a growth factor associated with angiogenesis and increased vascular permeability, often observed in the setting of inflammation, ischemia, or hypoxia [44]. VEGF also has neurotrophic and neuroprotective effects that play a homeostatic role in the brain during inflammation, ischemia/hypoxia, and other types of cell stress [44,45,46]. Increased VEGF has been associated with better treatment responses in depression and may play a key role in mediating the neurogenic and behavioral effects of various treatment modalities, including antidepressant drugs, electroconvulsive therapy (ECT), and repetitive transcranial magnetic stimulation [44,47,48,49]. These findings are consistent with our observation that subgroups of PWH with higher baseline plasma VEGF had greater improvement in depressive symptoms over time (Figure 2B).

Previous studies have detected increased blood VEGF levels in virally suppressed PWH on ART [37,50,51], but its biological significance in HIV pathogenesis and comorbid conditions remains unclear. The main source of VEGF in peripheral blood is activated platelets, which are a common finding in PWH that may be linked to chronic immune activation/inflammation despite ART. Our biomarker-driven clustering identified a subgroup of PWH with high depressive symptoms distinguished by high VEGF/IL-8/MCP-1, consistent with a model in which activated monocytes producing IL-8 and MCP-1 trigger VEGF release from activated platelets [52,53]. Activated monocytes and platelets have been observed in depression [7,13,53,54,55], chronic stress [56], and a variety of comorbid conditions that are prevalent among PWH, including HCV, CVD, diabetes, COPD, cirrhosis, end-stage kidney disease, and neurocognitive disorders [6,51,57]. One study detected increased CSF VEGF in HAND [58], while others detected increased plasma/serum VEGF in HAND [59] or MND [37] or reported decreased plasma VEGF-D (another member of the VEGF family, which is comprised of five members: VEGF-A, VEGF-B, VEGF-C, VEGF-D, and PGF) but no difference in VEGF in PWH with amnestic MCI [60]. Further studies are needed to understand the inter-relationships between increased blood VEGF levels, activated monocytes/platelets, and comorbid conditions in PWH.

Medical comorbidities were independently associated with depressive symptoms in our cohort of PWH on ART (Supplementary Table S2), consistent with prior studies [1,2,9,61,62]. Although the association between VEGF and depressive symptoms was augmented in PWH with medical comorbidities, VEGF levels were not significantly different between groups stratified by medical comorbidity status, and a higher prevalence of medical comorbidities among PWH with high vs. low depressive symptoms (72% vs. 57%) did not explain the group differences in VEGF levels. The medical comorbidities we examined have been associated with increased rates of depression in previous studies, irrespective of HIV [63,64]. Although we identified biomarker-defined subgroups distinguished by levels of VEGF/MCP-1/IL-8 or IL-6/CRP, their associated patterns of medical comorbidities were largely similar, with the exception of diabetes, which was more prevalent in the high VEGF/MCP-1/IL-8 cluster. Future studies of larger cohorts will be important to better define the relationships between inflammation biomarker patterns, specific comorbidities, and subtype(s) of depression in PWH on ART.

We acknowledge several limitations of the study, including the relatively small sample size, the under-representation of women (15%), and the heterogeneity of clinical characteristics such as medical comorbidities, substance use, and antidepressant use. Only a subset of PWH had available longitudinal BDI scores, which limited statistical power for mixed-effects models and analysis of subgroup trajectories. Depressive symptoms were assessed using BDI scores rather than clinical diagnoses because DSM-IV (*Diagnostic and Statistical Manual of Mental Disorders, Fourth Edition*, a manual used for classification of mental disorders based on specific diagnostic criteria) diagnoses were not available for a high proportion of the study participants. Thus, analyses based on clinical diagnoses may reveal different findings. BDI scores between 14 and 19 correspond to mild depressive symptoms. However, we did not have a sufficient sample size to perform analyses stratified by mild or moderate/severe depressive symptoms. Although we adjusted for the effects of antidepressants, we cannot exclude the possibility that the use of antidepressants or other medications influenced VEGF levels. We did not measure VEGF at more than one time point, so we cannot assess if any improvement in BDI scores was associated with a reduction in VEGF levels. Lastly, as this was an observational study, we could not determine causality. Prospective studies of larger cohorts are needed to better understand the prognostic value of VEGF and other biomarkers for predicting depressive symptoms and therapeutic responses.

In summary, we present evidence that VEGF is a stronger predictor of depressive symptoms than other inflammation markers in virally suppressed PWH on ART. These findings are consistent with previous studies that identified VEGF as a biomarker associated with major depressive disorder in the general population. The association between VEGF and depressive symptoms was augmented in PWH with medical comorbidities, suggesting a role for chronic medical conditions in shaping the pathophysiology of depressive disorders in this population. Increased plasma VEGF may reflect underlying inflammatory or vascular processes that alter CNS function, thereby contributing to depressive symptoms. Our findings, together with those of previous studies, suggest that VEGF may identify a subtype of depression for targeted interventions. For example, interventions targeting the VEGF pathway—including those that modulate monocyte/platelet activation, VEGF neurovascular signaling, or VEGF neurotrophic effects—may be particularly relevant for the VEGF-high inflammatory subtype and merit further investigation for PWH with this biomarker profile. Integrating peripheral blood VEGF measurements into clinical assessments of depression may facilitate stratification for testing tailored interventions and monitoring treatment response. Our findings also highlight the importance of accounting for virologic status (i.e., suppressed or viremic) and medical comorbidities in future studies to evaluate the associations between biomarkers and depression in PWH, as these factors can impact the interpretation of findings. Future studies will be important to determine the prognostic value of VEGF and associated inflammation biomarker patterns in the onset and treatment response of depression in PWH and to characterize inflammatory and noninflammatory subtypes of depression for the development of tailored therapies.

## Figures and Tables

**Table 1:** Characteristics of the study cohort by HIV status and depressive symptoms.

	HIV− Individuals (*n* = 65)	HIV+ Individuals (*n* = 195)	*p*-Value	HIV+ with Low Depressive Symptoms (*n* = 131)	HIV+ with High Depressive Symptoms (*n* = 64)	*p*-Value
Age (years)	58 [53, 65]	52 [47, 58]	<0.001	52 [47, 58]	51 [46, 58]	0.469
Race, *n* (%)						
Black	27 (42)	54 (28)	0.198	39 (30)	15 (23)	0.210
White	28 (43)	106 (54)		66 (50)	40 (63)	
Male sex, *n* (%)	39 (60)	165 (85)	0.001	111 (85)	54 (84)	1
BMI (kg/m^2^)	30 [25, 34]	25 [23, 28]	0.002	26 [23, 29]	24 [21, 26]	0.001
Current smoking, *n* (%)	44 (68)	105 (54)	0.070	68 (52)	37 (58)	0.533
Current cocaine use, *n* (%)	10 (15)	27 (14)	0.918	18 (14)	9 (14)	1
Duration of HIV infection (years)		16 [11, 21]		17 [11, 21]	15 [10, 20]	0.314
Plasma viral load < 200 copies/mL, *n* (%)		185 (95)		125 (95)	60 (94)	0.378
CD4+ T-cell count (cells/μL)		466 [307, 704]		437 [279, 692]	546 [404, 725]	0.104
Nadir CD4+ T-cell count (cells/μL)		66 [12, 191]		57 [10, 159]	96 [23, 229]	0.063
BDI score		8 [3, 17]		5 [1, 8]	22 [17, 27]	<0.001
Current antidepressant use, *n* (%)		86 (44)		47 (36)	39 (61)	0.002
Global cognitive T score		48 [42, 53]		48 [43, 53]	47 [41, 51]	0.158
HAND diagnosis, *n* (%)						
ANI		43 (22)		34 (26)	9 (14)	0.008
MND		30 (15)		18 (14)	12 (19)	
HAD		5 (3)		0 (0)	5 (8)	
NPI-O		23 (12)		16 (12)	7 (11)	
Normal		94 (48)		63 (48)	31 (48)	
Medical comorbidities, *n* (%)						
HCV Ab positive		57 (29)		34 (26)	23 (36)	0.204
Cardiovascular disease		47 (24)		27 (21)	20 (31)	0.146
Diabetes		26 (13)		19 (15)	7 (11)	0.643
CKD stage 3–4		18 (9)		10 (8)	8 (13)	0.402
COPD		39 (20)		22 (17)	17 (27)	0.158
≥1 Medical comorbidity		120 (62)		74 (57)	46 (72)	0.055
IL-1β (pg/mL)	0.11 [0.02, 0.20]	0.12 [0.05, 0.21]	0.139	0.11 [0.05, 0.20]	0.14 [0.07, 0.23]	0.145
IFN-γ (pg/mL)	18.2 [10.1, 29.9]	18.2 [10.3, 30.8]	0.817	17.5 [10.1, 28.9]	18.9 [10.4, 32.5]	0.607
IL-6 (pg/mL)	1.20 [0.80, 1.60]	1.10 [0.70, 1.85]	0.990	1.11 [0.72, 2.10]	0.99 [0.65, 1.53]	0.273
IL-8 (pg/mL)	3.3 [2.2, 5.7]	4.7 [3.2, 7.2]	0.001	4.6 [3.0, 7.2]	4.9 [3.3, 7.4]	0.489
IL-12p70 (pg/mL)	0.28 [0.14, 0.44]	0.31 [0.16, 0.45]	0.641	0.33 [0.18, 0.46]	0.28 [0.15, 0.44]	0.225
IL-15 (pg/mL)	4.2 [3.3, 5.8]	4.9 [3.7, 7.0]	0.037	5.1 [3.7, 7.1]	4.4 [3.7, 7.0]	0.555
IP-10 (pg/mL)	205 [140, 321]	346 [244, 549]	<0.001	317 [246, 551]	393 [240, 537]	0.527
MCP-1 (pg/mL)	78 [66, 102]	99 [77, 122]	0.001	97 [73, 119]	110 [86, 128]	0.041
VEGF (pg/mL)	24 [12, 48]	48 [32, 75]	<0.001	45 [31, 66]	64 [34, 100]	0.001
CRP (ug/mL)	2.5 [1.3, 9.1]	3.4 [2.3, 6.4]	0.042	3.4 [2.2, 6.4]	3.4 [2.4, 6.8]	0.619

Medians [interquartile range] are shown unless otherwise indicated. *p*-values for two-group comparisons between HIV− vs. HIV+ or between HIV+ with high vs. low depressive symptoms were calculated using Chi-square test for categorical variables and Mann–Whitney U test for continuous variables. Depressive symptoms and medical comorbidities were defined as described in the Methods. Abbreviations: Ab, antibody; ANI, asymptomatic neurocognitive impairment; BDI, Beck Depression Inventory-II (BDI-II); BMI, body mass index; CKD, chronic kidney disease; COPD, chronic obstructive pulmonary disease; HAD, HIV-associated dementia; HAND, HIV-associated neurocognitive disorder; MND, mild neurocognitive disorder; NPI-O, neuropsychological impairment attributable to other causes.

**Table 2:** Association between VEGF and depressive symptoms is augmented in HIV+ individuals with medical comorbidities.

	HIV+ Individuals (*n* = 195)				HIV+ Without Medical Comorbidities (*n* = 75)		HIV+ with Medical Comorbidities (*n* = 120)	
	Univariate Models		Multivariable Model		Multivariable Model		Multivariable Model	
Predictor	OR (95% CI)	*p*-Value	OR (95% CI)	*p*-Value	OR (95% CI)	*p*-Value	OR (95% CI)	*p*-Value
Log10 VEGF	**4.02 (1.47, 11.6)**	**0.008**	**4.97 (1.65, 15.8)**	**0.005**	2.19 (0.19, 27.1)	0.529	**7.63 (2.16, 30.2)**	**0.002**
Age	0.88 (0.61, 1.25)	0.466	0.77 (0.51, 1.14)	0.195	0.55 (0.23, 1.23)	0.158	0.83 (0.51, 1.35)	0.460
Male sex	0.97 (0.43, 2.30)	0.948	0.89 (0.35, 2.32)	0.804	0.51 (0.09, 3.06)	0.447	1.35 (0.42, 4.71)	0.619
BMI	**0.88 (0.81, 0.95)**	**0.001**	**0.87 (0.80, 0.94)**	**0.001**	**0.84 (0.70, 0.97)**	**0.032**	**0.89 (0.81, 0.98)**	**0.019**
Antidepressant use	**2.79 (1.52, 5.21)**	**0.001**	**2.72 (1.42, 5.28)**	**0.003**	**4.40 (1.32, 16.5)**	**0.020**	2.17 (0.98, 4.99)	0.062

Logistic regression models with high vs. low depressive symptoms as the dependent variable were fit for each predictor among all 195 HIV+ individuals, 75 HIV+ individuals without medical comorbidities, and 120 HIV+ individuals with 1 or more medical comorbidities. Log10 VEGF is independently associated with high depressive symptoms in multivariable models adjusted for age, sex, BMI, and current antidepressant use. High depressive symptoms and medical comorbidities were defined as described in the Methods. Abbreviations: BMI, body mass index; CI, confidence interval; OR, odds ratio. Bold denotes *p* < 0.05.

**Table 3:** Longitudinal association between VEGF tertile and BDI score over 18 months of follow-up.

	HIV+ Individuals (*n* = 140)				HIV+ Without Medical Comorbidities (*n* = 56)		HIV+ with Medical Comorbidities (*n* = 84)	
	Time Adjusted Models		Covariate and Time Adjusted Model		Covariate and Time Adjusted Model		Covariate and Time Adjusted Model	
Predictor	Estimate (95% CI)	*p*-Value	Estimate (95% CI)	*p*-Value	Estimate (95% CI)	*p*-Value	Estimate (95% CI)	*p*-Value
VEGF (high tertile)	**3.47 (0.22, 6.74)**	**0.039**	3.19 (−0.01, 6.38)	0.056	2.22 (−0.29, 7.35)	0.418	**4.08 (0.15, 8.01)**	**0.050**
Age	−0.05 (−0.22, 0.13)	0.593	−0.01 (−0.18, 0.15)	0.869	−0.24 (−0.55, 0.07)	0.148	0.05 (−0.15, 0.25)	0.640
Male sex	−3.99 (−8.37, 0.41)	0.076	−3.76 (−8.16, 0.63)	0.100	−4.99 (−11.39, 1.39)	0.146	−1.31 (−7.17, 4.54)	0.669
BMI	−0.23 (−0.51, 0.05)	0.111	**−0.31 (−0.59, −0.04)**	**0.031**	−0.44 (−0.88, −0.01)	0.061	−0.26 (−0.61, 0.08)	0.144
Antidepressant use	**4.13 (1.12, 7.13)**	**0.007**	**3.69 (0.78, 6.60)**	**0.015**	**6.47 (1.66, 11.26)**	**0.012**	1.76 (−1.81, 5.32)	0.348

Linear mixed-effects models with BDI score as the dependent variable were fit for each predictor among 140 HIV+ individuals, 56 HIV+ individuals without medical comorbidities, and 84 HIV+ individuals with 1 or more medical comorbidities and 2 or more BDI scores available within 18 months of follow-up. VEGF (high tertile vs. middle/low tertiles) is independently associated with depressive symptoms in mixed-effects models adjusted for age, sex, BMI, current antidepressant use, and time (months). Plots are shown in Figure 1. Medical comorbidities were defined as described in the Methods. Abbreviations: BDI, Beck Depression Inventory-II (BDI-II); BMI, body mass index; CI, confidence interval. Bold denotes *p* ≤ 0.05.

## Data Availability

All data generated or analyzed during this study are included in the published article and its Supplementary Information files or available from the corresponding author upon reasonable request.

## Supplementary Materials

The following supporting information can be downloaded at https://www.mdpi.com/article/10.3390/v18060628/s1: Figure S1: Correlation analysis of VEGF versus MCP-1 levels among all 195 HIV+ individuals or subset of 64 HIV+ individuals with high depressive symptoms; Figure S2: Contribution of each of the 10 inflammation biomarkers to 1st and 2nd principal components (Dim1 and Dim2); Table S1: Association between MCP-1 and depressive symptoms is attenuated in models adjusted for VEGF levels; Table S2: Association between ≥1 medical comorbidity and depressive symptoms remains significant in models adjusted for VEGF; Table S3: Demographic, clinical, and biomarker characteristics of HIV+ individuals with high depressive symptoms according to biomarker clusters.
