# Supplementary material for: VEGF Is a Stronger Predictor of Depressive Symptoms than Other Inflammation Markers in People with HIV on Antiretroviral Therapy
Source: Viruses. 2026 May 30;18(6):628. doi: 10.3390/v18060628 (PMC13307925; doi:10.3390/v18060628)
Supplement: Supplementary file 1 [file viruses-18-00628-s001.zip › Supplementary Figures S1 and S2.pdf]

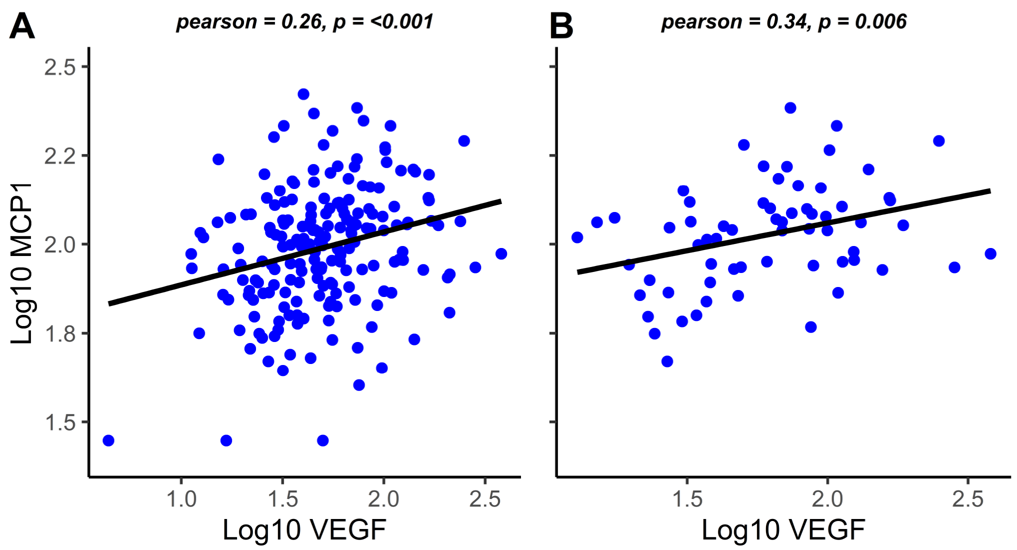

Supplementary Figure S1. Correlation analysis of VEGF versus MCP-1 levels among all 195 HIV+ individuals or subset of 64 HIV+ individuals with high depressive symptoms. (A) Correlation between log10 VEGF and log10 MCP-1 (pg/ml) among 195 HIV+ individuals. (B) Correlation analysis as described in (A) restricted to 64 HIV+ individuals with high depressive symptoms. Pearson's r and p-values are shown.

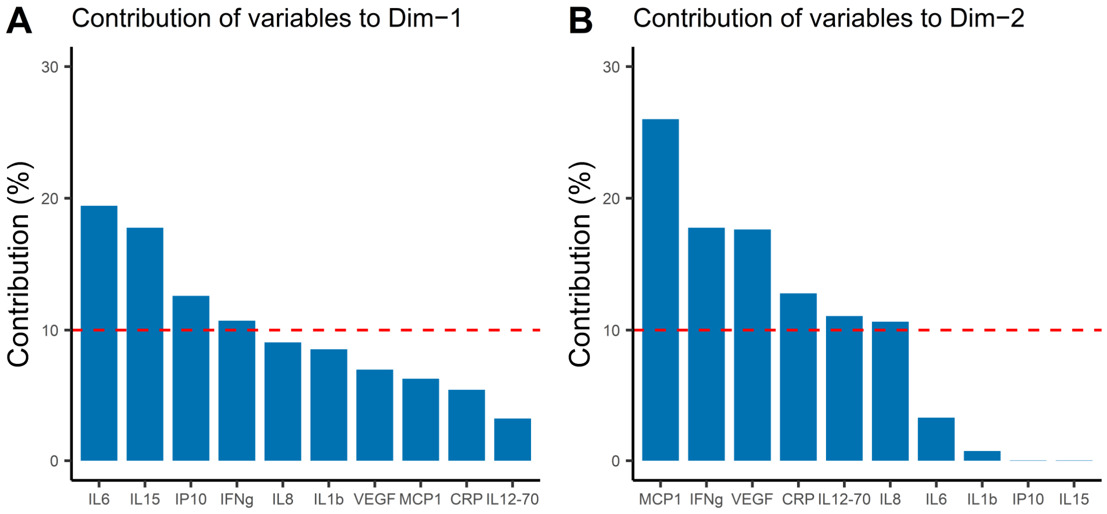

Supplementary Figure S2. Contribution of each of the 10 inflammation biomarkers to 1st and 2nd principal components (Dim1 and Dim2). Bar charts show the contribution of each biomarker (%) to Dim1 (panel A) and Dim2 (panel B). The total sum of contributions is 100% for each principal component. The red dashed line indicated the expected average contribution of each biomarker, i.e., 100%/10 = 10%.
