# Supplementary material for: VEGF Is a Stronger Predictor of Depressive Symptoms than Other Inflammation Markers in People with HIV on Antiretroviral Therapy
Source: Viruses. 2026 May 30;18(6):628. doi: 10.3390/v18060628 (PMC13307925; doi:10.3390/v18060628)
Supplement: Supplementary file 1 [file viruses-18-00628-s001.zip › Supplementary Tables S1-S3.pdf]

SUPPLEMENTARY TABLE 1. Association between MCP-1 and depressive symptoms is attenuated in models adjusted for VEGF levels.

	**Univariate models**		**Multivariable model MCP-1+Age+Sex+BMI+Antidepressant**		**Multivariable model MCP-1+VEGF+Age+Sex+BMI+Antidepressant**	
**Predictor**	**OR (95% CI)**	***p*-value**	**OR (95% CI)**	***p*-value**	**OR (95% CI)**	***p*-value**
Log10 MCP-1	**6.54 (1.10, 42.83)**	**0.043**	**10.25 (1.39, 87.16)**	**0.027**	6.63 (0.84, 58.58)	0.078
Log10 VEGF	**4.02 (1.47, 11.60)**	**0.008**			**4.23 (1.36, 13.71)**	**0.014**
Age	0.88 (0.61, 1.25)	0.466	0.77 (0.51, 1.14)	0.193	0.69 (0.44, 1.04)	0.084
Male sex	0.97 (0.43, 2.30)	0.948	0.70 (0.28, 1.83)	0.460	0.82 (0.32, 2.18)	0.682
BMI	**0.88 (0.81, 0.95)**	**0.001**	**0.86 (0.81, 0.94)**	**0.001**	**0.87 (0.80, 0.94)**	**0.001**
Antidepressant use	**2.79 (1.52, 5.21)**	**0.001**	**2.75 (1.45, 5.32)**	**0.002**	**2.69 (1.40, 5.25)**	**0.003**

Logistic regression models with high vs. low depressive symptoms as the dependent variable were fit for each predictor among 195 HIV+ individuals in models adjusting for age, sex, BMI, and antidepressant use with and without adjusting for Log10 VEGF. High depressive symptoms and medical comorbidities were defined as described in the Methods. Abbreviations: BMI, body mass index; CI, confidence interval; OR, odds ratio. Bold denotes $p < 0.05$.

SUPPLEMENTARY TABLE 2. Association between >1 medical comorbidity and depressive symptoms remains significant in models adjusted for VEGF.

	**Univariate models**		**Multivariable model Comorbidity+Age+Sex +BMI+Antidepressant**		**Multivariable model Comorbidity+VEGF+Age+Sex +BMI+Antidepressant**	
**Predictor**	**OR (95% CI)**	***p*-value**	**OR (95% CI)**	***p*-value**	**OR (95% CI)**	***p*-value**
>1 Medical comorbidity	1.64 (0.90, 3.05)	0.112	**2.00 (1.02, 4.00)**	**0.047**	**2.15 (1.08, 4.41)**	**0.032**
Log10 VEGF	**4.02 (1.47, 11.60)**	**0.008**			**5.31 (1.76, 16.99)**	**0.004**
Age	0.88 (0.61, 1.25)	0.466	0.81 (0.54, 1.19)	0.277	0.68 (0.45, 1.03)	0.075
Male sex	0.97 (0.43, 2.30)	0.948	0.77 (0.31, 1.96)	0.581	0.88 (0.35, 2.32)	0.793
BMI	**0.88 (0.81, 0.95)**	**0.001**	**0.88 (0.81, 0.94)**	**0.001**	**0.87 (0.80, 0.94)**	**0.001**
Antidepressant use	**2.79 (1.52, 5.21)**	**0.001**	**2.96 (1.56, 5.75)**	**0.001**	**2.90 (1.50, 5.74)**	**0.002**

Logistic regression models with high vs. low depressive symptoms as the dependent variable were fit for each predictor among 195 HIV+ individuals in models adjusting for age, sex, BMI, antidepressant use, and >1 medical comorbidity with and without adjusting for Log10 VEGF. High depressive symptoms and medical comorbidities were defined as described in the Methods. Abbreviations: BMI, body mass index; CI, confidence interval; OR, odds ratio. Bold denotes $p < 0.05$.

SUPPLEMENTARY TABLE 3. Demographic, clinical, and biomarker characteristics of HIV+ individuals with high depressive symptoms according to biomarker clusters

Variable	Cluster 1 (n = 26)	Cluster 2 (n = 26)	Cluster 3 (n = 11)	p-value
Age (years)	53.9 [49.7, 59.2]	49.6 [43.6, 54.3]	48.4 [43.7, 53.9]	0.0195
Race, n (%)				
Black	7 (26.9)	3 (11.5)	4 (36.4)	0.1964
White	17 (65.4)	18 (69.2)	5 (45.5)	
Male sex, n (%)	24 (92.3)	23 (88.5)	6 (54.5)	0.0118
BMI (kg/m2)	24.4 [22.6, 26.5]	23.7 [21.1, 26.4]	22.8 [20.9, 24.2]	0.4629
Current smoking, n (%)	15 (57.7)	12 (46.2)	9 (81.8)	0.134
Current cocaine use, n (%)	2 (7.7)	3 (11.5)	4 (36.4)	0.0652
Duration of HIV infection (years)	17.4 [13.2, 22.2]	15.2 [8.8, 19.9]	13.7 [10.1, 14.4]	0.1593
Plasma viral load (copies/mL)	40 [40, 57]	40 [40, 40]	40 [40, 40]	0.1514
CD4+ T-cell count (cells/µL)	580 [406, 705]	551 [416, 720]	442 [284, 649]	0.5039
Nadir CD4+ T-cell count (cells/µL)	130 [41, 237]	95 [8, 215]	28 [11, 209]	0.4386
BDI score	23 [19, 28]	19 [17, 23]	26 [21, 32]	0.0922
Current antidepressant use, n (%)	14 (53.8)	18 (69.2)	7 (63.6)	0.5164
Global cognitive T score	47.56 [42.38, 50.74]	47.58 [42.47, 54.08]	37.20 [27.62, 47.69]	0.1357
HAND diagnosis, n (%)				
ANI	3 (11.5)	4 (15.4)	2 (18.2)	0.0002
MND	7 (26.9)	4 (15.4)	0 (0.0)	
HAD	0 (0.0)	0 (0.0)	5 (45.5)	
NPI-O	4 (15.4)	2 (7.7)	1 (9.1)	
Normal	12 (46.2)	16 (61.5)	3 (27.3)	
Medical comorbidities, n (%)				
HCV Ab positive	12 (46.2)	5 (19.2)	5 (45.5)	0.0909
Cardiovascular disease	10 (38.5)	4 (15.4)	5 (45.5)	0.0922
Diabetes	6 (23.1)	1 (3.8)	0 (0.0)	0.0381
CKD stage 3-4	3 (11.5)	2 (7.7)	2 (18.2)	0.6475
COPD	9 (34.6)	4 (15.4)	4 (36.4)	0.2192
$\geq$ 1 Medical comorbidity	22 (84.6)	14 (53.8)	9 (81.8)	0.0345
IL-1b (pg/ml)	0.14 [0.11, 0.20]	0.09 [0.02, 0.22]	0.21 [0.13, 0.30]	0.0987
IFN-g (pg/ml)	20.53 [10.61, 29.47]	13.31 [7.87, 19.11]	58.12 [39.29, 73.23]	<0.0001
IL-6 (pg/ml)	1.28 [0.89, 1.57]	0.67 [0.50, 0.89]	2.07 [1.43, 3.93]	<0.0001
IL-8 (pg/ml)	7.11 [5.14, 9.17]	3.27 [2.61, 4.48]	4.65 [3.51, 5.19]	<0.0001
IL-12p70 (pg/ml)	0.29 [0.16, 0.43]	0.24 [0.05, 0.36]	0.44 [0.27, 0.55]	0.074
IL-15 (pg/ml)	6.63 [4.69, 7.78]	3.60 [3.00, 4.03]	5.84 [4.03, 8.85]	<0.0001
IP-10 (pg/ml)	456.80 [264.55, 807.04]	280.86 [208.17, 410.25]	483.19 [397.52, 674.00]	0.0031
MCP-1 (pg/ml)	131.96 [116.05, 164.71]	89.25 [80.71, 109.22]	93.91 [72.30, 115.17]	<0.0001
VEGF (pg/ml)	82.18 [60.72, 121.33]	39.31 [26.91, 67.28]	47.99 [34.39, 98.00]	0.0028
CRP (ug/ml)	2.85 [2.26, 3.47]	3.47 [2.40, 5.78]	15.63 [7.03, 24.63]	0.0001

Median (interquartile range) are shown unless otherwise indicated. P-values for comparisons across groups of HIV+ individuals with high depressive symptoms according to biomarker-defined clusters were calculated using the chi-square test for categorical variables and Kruskal-Wallis test for continuous variables. Because the 10 biomarkers were used to define the clusters by K-means, the across-cluster differences in biomarker levels reflect the clustering input rather than independent statistical evidence; p-values are descriptive only. Depressive symptoms and medical comorbidities were defined as described in the Methods. Abbreviations: Ab, antibody; ANI, asymptomatic neurocognitive impairment; BDI, Beck Depression Inventory-II (BDI-II); BMI, body mass index; CKD, chronic kidney disease; COPD, chronic obstructive pulmonary disease; HAD, HIV-associated dementia; HAND, HIV-associated neurocognitive disorder; MND, mild neurocognitive disorder; NPI-O, neuropsychological impairment attributable to other causes.
